# Preliminary phylogenetic analysis of the Andean clade and the placement of new Colombian blueberries (Ericaceae, Vaccinieae)

**DOI:** 10.3897/phytokeys.49.8622

**Published:** 2015-04-22

**Authors:** Paola Pedraza-Peñalosa, Nelson R. Salinas, Anne Lucy S. Virnig, Ward C. Wheeler

**Affiliations:** 1 The New York Botanical Garden, Institute of Systematic Botany, Bronx, NY 10458, U.S.A.; 2 City University of New York, The Graduate Center, 365 Fifth Avenue, New York, NY 10016, U.S.A.; 3 American Museum of Natural History, Division of Invertebrate Zoology, Central Park West at 79th Street, New York, NY 10024, U.S.A.

**Keywords:** Ericaceae, Vaccinieae, Andes, Molecular phylogeny, New species, Colombia

## Abstract

The blueberry tribe Vaccinieae (Ericaceae) is particularly diverse in South America and underwent extensive radiation in Colombia where many endemics occur. Recent fieldwork in Colombia has resulted in valuable additions to the phylogeny and as well in the discovery of morphologically noteworthy new species that need to be phylogenetically placed before being named. This is particularly important, as the monophyly of many of the studied genera have not been confirmed. In order to advance our understanding of the relationships within neotropical Vaccinieae and advice the taxonomy of the new blueberry relatives, here we present the most comprehensive phylogenetic analysis for the Andean clade. *Anthopterus*, *Demosthenesia*, and *Pellegrinia* are among the putative Andean genera recovered as monophyletic, while other eight Andean genera were not. The analyses also showed that genera that have been traditionally widely defined are non-monophyletic and could be further split into more discrete groups. Four newly discovered Colombian Vaccinieae are placed in the monophyletic *Satyria* *s.s.* and the *Psammisia* I clade. Although these new species are endemic to the Colombian Western Cordillera and Chocó biogeographic region and three are not known outside of Las Orquídeas National Park, they do not form sister pairs.

## Introduction

In the neotropical regions, the most extensive radiation of the plant family Ericaceae took place in Colombia where there are 24 genera and 278 described species. Notably, about 55% of the Colombian Ericaceae are endemic to the country (Pedraza-Peñalosa unpubl.). Within Colombia, the greatest documented diversity of Ericaceae is found in the Western Cordillera and adjacent Chocó region, which belong to the Tropical Andes and Chocó biodiversity hotspots (Mittermeier et al. 1998; Myers et al. 2000), respectively. These hotspots have the highest angiosperm diversity in N South America (Morawetz and Raedig 2007), but despite their importance for the understanding of the genesis of the neotropical flora, entire lineages particularly rich in NW Colombia are missing in modern monographic and phylogenetic research, and not only in Ericaceae.

Over the past 40 years, major U.S. herbaria have observed a sharp decline (ca. > 85%; calculated between 1970–2009) in the number of Colombian specimens received and databased. This decline reflects the decrease in field-based projects and exchange of herbarium specimens due to a combination of safety concerns, complicated Colombian permitting legislation, and lack of funding.

It is only recently that Colombian Ericaceae are being included in molecular analyses (see *Disterigma* (Klotzsch) Nied. in Pedraza-Peñalosa 2009, 2010a, 2010b). The ongoing inventory of the vascular plants of Las Orquídeas National Park (LONP), strategically located in the confluence of the Colombian Tropical Andes and Chocó regions, has made available interesting new material of Ericaceae. LONP is a poorly known and isolated protected area in NW Antioquia within the general region with the greatest documented diversity and endemism of Ericaceae in the Neotropics (Luteyn 2002). Four new members of the tribe Vaccinieae stand out among the many other new plant species discovered in LONP. All of them are endemic to the Colombian Western Cordillera and Chocó biogeographic region, and three of them are endemic to LONP. Vaccinieae include the edible North American blueberries and South American *mortiños* and make up the bulk of Ericaceae in Colombia.

There are about 600 species of Vaccinieae in the Neotropics, currently placed in 30 genera, 28 of them endemic to the region (Luteyn 2002). Large-scale phylogenetic analyses of Vaccinieae are few. In a preliminary analysis of the entire tribe, Kron et al. (2002) found that the great majority of the neotropical taxa are resolved within an Andean clade. This clade has about 500 species (Pedraza-Peñalosa unpubl.) and includes species growing as far south as Bolivia. The only study of the N Andean blueberries is one that included 55 species and 14 Andean genera (Powell and Kron 2003). However, because Colombian taxa have been largely unavailable, Colombian species were largely absent from these studies.

Kron et al. (2002) and Powell and Kron (2003) are studies with different scopes, but both found a striking disparity between phylogenetic relationships and the current classification system, with 60–80% of the genera sampled resolved as not monophyletic. However, because tropical Ericaceae are very diverse, hard to collect and sometimes hard to sequence, phylogenetic analyses comprehensive enough to readdress generic limits are not available yet. In the absence of a phylogenetic based classification for Vaccinieae, the generic placement of novel species remains challenging, at best.

Morphology is most frequently the only tool available to determine the taxonomic identity of a new Vaccinieae and the morphology of the four new species from LONP place them in *Satyria* Klotzsch and *Psammisia* Klotzsch, both broadly-circumscribed groups that have been shown to be non-monophyletic. *Satyria* and *Psammisia* are both placed by nuclear and chloroplast molecular data within the Andean clade (Kron et al. 2002, Powell and Kron 2003). *Psammisia*, the second largest neotropical Ericaceae genus, is paraphyletic with respect to *Macleania* Hook., while the small-sized *Satyria* is polyphyletic. While the phylogenetic relationships of *Psammisia* and *Macleania* have not been addressed in more detail, molecular analyses revealed that the species of *Satyria* are placed in two clades that are not closely related to each other. *Satyria* from Central America and N South America are congeneric and form *Satyria* s.s. (including the type species, *Satyria warszewiczii* Klotzsch), whereas species of *Satyria* from S Peru and Bolivia cluster with representatives of *Thibaudia* Ruiz & Pav. ex J. St.-Hil. from the same geographic region, forming the *Thibaudia* clade, which is placed in a distant part of the phylogenetic tree (Powell and Kron 2003). All the previously mentioned groups, except for *Thibaudia*, are most diverse in N South America.

The objectives of this study are twofold, first, to provide the most comprehensive phylogenetic analysis yet for the entire Andean clade, second, to discover the evolutionary affinities of the novel taxa from LONP. These phylogenetic results will be used to guide their future naming. Although special emphasis has been placed in the representation of the neglected Colombian taxa, making of this dataset the largest published for neotropical Vaccinieae, more work is still necessary to elucidate intergeneric relationships within the Andean clade. Consequently, our results are still only preliminary, but they lay the groundwork for future detailed studies within and across Andean Vaccinieae. Lastly, although no attempt to reconstruct the evolution of morphological characters is made here, the morphology associated with the best-supported clades is briefly discussed when relevant.

## Material and methods

### Taxon sampling

Sequence data from 94 terminals (91 species), belonging to 20 putatively neotropical Vaccinieae genera, were analyzed. The sampling strategy followed that of Pedraza-Peñalosa et al. (2013), but with emphasis on taxa of Andean origin. Eighteen species endemic to Colombia were newly sequenced and for five other species that also grow outside Colombia, a population from Colombia was chosen. The sampled taxa exemplify different aspects of reproductive and vegetative morphology. They also represent the major clades recovered within Neotropical Vaccinieae in previous phylogenetic analyses, and also include species from the Caribbean/Mesoamerican clade. Emphasis was placed on sampling *Satyria*, *Macleania*, *Psammisia*, *Thibaudia* and *Cavendishia* Lindl. For polymorphic species hard to identify or those whose variation is insufficiently known, more than one specimen was sequenced. For the plastic *Satyria grandifolia* Hoerold, two specimens recently collected in LONP (NW Colombia), each representing a separate morphospecies, were sequenced. These were analyzed along with the sequence already available in GenBank and originally collected in SW Colombia. Two specimens were also sequenced of the very rare and morphologically insufficiently understood *Psammisia mediobullata* Luteyn & Sylva, endemic to a small region of NW Antioquia (Colombia). Trees were rooted with *Gaylussacia baccata* (Wangenh.) K.Koch, a species from a genus of extra-neotropical origin that is basal with respect to all New World taxa (Kron et al. 2002).

### DNA extraction and sequencing

A combination of nuclear (nrITS, 651 aligned bp.) and plastid (5’ end of *ndhF*, ca. 1225 aligned bp.; *matK*, 1331 aligned bp.) markers were selected because of their number of phylogenetically informative characters in previous studies (Kron et al. 2002, Powell and Kron 2003, Pedraza-Peñalosa 2009, 2010a, Pedraza-Peñalosa et al. 2013). All procedures used during the DNA extraction and sequencing have been published previously (Pedraza-Peñalosa 2009). Sequences were edited with Sequencher 5.2.3 (Gene Codes Corporation). For this study, 93 new molecular sequences were produced (permits DTSA 033 SFF Galeras y otros; Acceso a Recursos Genéticos Res. 734 de 30 de Abril de 2007; 35-2005-INRENA-IFFS-DCB), the rest were gathered from GenBank; all accession numbers are provided in Table 1.

**Table 1.** Species of Vaccinieae studied with their corresponding voucher specimens and GenBank accession numbers (ITS, *matK*, *ndhF*). m = missing, RBGE = Royal Botanic Garden Edinburgh, RBGK = Royal Botanic Garden Kew, LONP = Las Orquídeas National Park.

Species	DNA sample (GenBank)
*Anthopterus revolutus* (Wilbur & Luteyn) Luteyn	Powell 20 (AY331866, AY331893, AY331920)
*Anthopterus wardii* Ball	Luteyn 15191 (AF382656, AF382746, AY331921)
*Cavendishia grandifolia* Hoerold (Subg. *Cavendishia*, sect. *Engleriana*, ser. *Engleriana*)	Luteyn 8023(AY331869, AY331896, AY331924)
*Cavendishia adenophora* Mansf. (Subg. *Cavendishia*, sect. *Engleriana*, ser. *Engleriana*)	Pedraza 1709 (KJ788222, KJ788253, KJ788191)
*Cavendishia angustifolia* Mansf. (Subg. *Cavendishia*, sect. *Engleriana*, ser. *Engleriana*)	Pedraza 1769 (KJ788223, KJ788254, KJ788192)
*Cavendishia bomareoides* A.C.Sm. (Subg. *Cavendishia*, sect. *Callista*)	Pedraza 1752 (KJ788224, KJ788255, KJ788193)
*Cavendishia bracteata* (Ruiz & Pav. ex J.St.-Hil.) Hoerold (Subg. *Cavendishia*, sect. *Cavendishia*, ser. *Cavendishia*)	Luteyn 14223 (AY331867, AY331894, AY331922)
*Cavendishia capitulata* Donn.Sm. (Subg. *Cavendishia*, sect. *Cavendishia*, ser. *Cavendishia*)	Powell 10 (AY331868, AY331895, AY331923)
Cavendishia complectens Hemsl. subsp. striata (A. C. Smith) Luteyn var. cylindrica Luteyn (Subg. *Cavendishia*, sect. *Cavendishia*, ser. *Imbricatae*)	Pedraza 1749 (KJ788225, KJ788256, KJ788194)
*Cavendishia leucantha* Luteyn (Subg. *Cavendishia*, sect. *Cavendishia*, ser. *Deciduae*)	Pedraza 1768 (KJ788226, KJ788257, KJ788195)
*Cavendishia lindauiana* Hoerold (Subg. *Cavendishia*, sect. *Callista*)	Pedraza 1766 (KJ788227, KJ788258, KJ788196)
*Cavendishia martii* (Meisn.) A.C.Sm. (Subg. *Cavendishia*, sect. *Quereme*)	Luteyn 15443 (AF382658, AF382747, AY331925)
*Cavendishia micayensis* A.C.Sm. (Subg. *Chlamydantha*)	Pedraza 1888 (KJ788228, KJ788259, KJ788197)
*Cavendishia pilosa* Luteyn (Subg. *Cavendishia*, sect. *Cavendishia*, ser. *Cavendishia*)	Pedraza 1743 (KJ788229, KJ788260, KJ788198)
*Cavendishia pubescens* (Kunth) Hemsl. (Subg. *Cavendishia*, sect. *Cavendishia*, ser. *Cavendishia*)	Pedraza 1038 (KJ788230, KJ788261, KJ788199)
*Cavendishia quereme* (Kunth) Benth. & Hook. f. (Subg. *Cavendishia*, sect. *Quereme*)	Pedraza 1707 (KJ788231, KJ788262, KJ788200)
Cavendishia tarapotana (Meisn.) Benth. & Hook. f. var. tarapotana Luteyn (Subg. *Cavendishia*, sect. *Cavendishia*, ser. *Cavendishia*)	Pedraza 1958 (KJ788232, KJ788263, KJ788201)
*Cavendishia tryphera* A.C.Sm. (Subg. *Cavendishia*, sect. *Engleriana*, ser. *Engleriana*)	Pedraza 1702 (KJ788233, KJ788264, KJ788202)
*Ceratostema lanceolatum* Bentham	Luteyn 15107 (AF382660, AF382749, m)
*Ceratostema lanigerum* (Sleumer) Luteyn	Luteyn 14216 (AY331870, AY331897, AY331926)
*Ceratostema megabracteatum* Luteyn	Luteyn 15037 (AF382661, AF382750, m)
*Ceratostema rauhii* Luteyn	Rauh 68468 (AY331871, AY331898, AY331927)
*Ceratostema reginaldii* (Sleumer) A.C.Sm.	Luteyn 14159 (AY331872, AY331899, AY331928)
*Ceratostema silvicola* A.C.Sm.	ABG 90-1101 (=Pedraza 1021) (AY331873, AY331900, AY331929)
*Demosthenesia mandonii* (Britton) A.C.Sm.	Luteyn 15433 (AF382664, AF382751, m)
*Demosthenesia spectabilis* (Rusby) A.C.Sm.	Luteyn 15474 (AF382665, AF382753, m)
*Diogenesia alstoniana* Sleumer	Luteyn 15196 (AF382672, AF382759, m)
*Diogenesia racemosa* (Herzog) Sleumer	Luteyn 15462 (AF382673, AF382760, AY331931)
*Disterigma agathosmoides* (Wedd.) Nied.	Pedraza 1001/Luteyn 15191 (FJ001671, KC175470, FJ001710)
*Disterigma pentandrum* S.F.Blake	Pedraza 1201/Luteyn 15085 (FJ001693, KC175465, FJ001733)
*Disterigma pseudokillipiella* Luteyn	Pedraza 1143, 1066 (FJ001694, KC175471, FJ001735)
*Disterigma rimbachii* (A.C.Sm.) Luteyn	Pedraza 1018 (FJ001695, KC175463, FJ001736)
*Disterigma trimerum* Wilber & Luteyn	Luteyn 15568 (FJ001700, KC175464, FJ001741)
*Gaylussacia baccata* K.Koch	Floyd 858 (AF273713, m, m)
*Gonocalyx costaricensis* Luteyn	Luteyn 15228 (AF382678, AF382764, m)
*Gonocalyx megabracteolatus* (Wilbur & Luteyn) Luteyn	Luteyn 14817 (AF382682, AF382767, m)
*Macleania bullata* Yeo	Luteyn 15724 (AF382679, U89758, AY331937)
*Macleania coccoloboides* A.C.Sm.	Luteyn 15852A (AF382680, AF382765, AY331938)
*Macleania cordifolia* Benth.	Pedraza 1884 (AY331877, AY331904, AY331939)
*Macleania floribunda* Hook.	Pedraza 1882 (FJ001704, m, FJ001745)
*Macleania insignis* M. Martens & Galeotti	RBGK 1969-19236 (AF382681, AF382766, AY331940)
*Macleania rupestris* (Kunth) A.C.Sm.	Pedraza 1879 (KC175462, m, KC175457)
*Notopora schomburgkii* Hook.f.	Luteyn 15275 (AF382683, AF382768, AF419728)
*Orthaea apophysata* (Griseb.) A.C.Sm.	van der Kloet 37694 (AF382685, AF382770, m)
*Orthaea venamensis* Maguire, Steyermark & Luteyn	Luteyn 15277 (AF382687, AF382772, m)
*Pellegrinia coccinea* (Hoerold) Sleumer	Luteyn 15646 (KC175461, KC175468, KC175453)
*Pellegrinia hirsuta* (Ruiz & Pav. ex G.Don) Sleumer	Luteyn 15644 (KC175458, KC175466, KC175455)
*Psammisia aberrans* A.C.Sm.	Pedraza 1715 (KJ788234, KJ788265, KJ788203)
*Psammisia breviflora* (Benth.) Klotzsch	Pedraza 2133 (KJ788235, KJ788266, KJ788204)
*Psammisia dolichopoda* A.C.Sm.	Luteyn 15006 (AF382690, AF382775, AF419730)
*Psammisia ecuadorensis* Hoerold	Luteyn 15033 (AF382691, AF382776, AY331942)
*Psammisia ferruginea* A.C.Sm.	Pedraza 1706 (KJ788237, KJ788268, KJ788206)
*Psammisia grandiflora* Hoerold	Pedraza 1101 (KJ788238, KJ788269, KJ788207)
*Psammisia* sp. nov. 1	Salinas 865 (KJ788243, KJ788274, KJ788212)
*Psammisia* sp. nov. 2	Pedraza 2134 (KJ788244, KJ788275, KJ788213)
*Psammisia mediobullata* Luteyn & Sylva “PP 2005”	Pedraza 2005 (KJ788239, KJ788270, KJ788208)
*Psammisia mediobullata* Luteyn & Sylva “PP 2129”	Pedraza 2129 (KJ788240, KJ788271, KJ788209)
*Psammisia oreogenes* Sleumer	Betancur 12349 (KJ788236, KJ788267, KJ788205)
*Psammisia pedunculata* A.C.Sm.	Pedraza 1754 (KJ788241, KJ788272, KJ788210)
*Psammisia ramiflora* Klotzsch	Setaro 08M33 (KJ788242, KJ788273, KJ788211)
*Psammisia sodiroi* Hoerold	Luteyn 8021 (AY331878, AY331905, AY331943)
*Psammisia ulbrichiana* Hoerold	Luteyn 15170 (AY331879, AY331906, AY331944)
*Satyria allenii* A.C.Sm.	Luteyn 15292 (AF382692, AF382777, AY331945)
*Satyria arborea* A.C.Sm.	Pedraza 1741 (KJ788245, KJ788276, KJ788214)
*Satyria boliviana* Luteyn	Luteyn 15481 (AF382693, AF382778, AY331946)
*Satyria bracteolosa* A.C.Sm.	Pedraza 2411 (KJ788246, KJ788277, KJ788215)
*Satyria cerander* (Dunal) A.C.Sm.	Mori 25279 (AY331880, AY331907, AY331947)
*Satyria grandifolia* Hoerold	Luteyn 15204 (AF382694, AF382779, AY331948)
*Satyria grandifolia* Hoerold “PP 2350”	Pedraza 2350 (KJ788247, KJ788278, KJ788216)
*Satyria grandifolia* Hoerold “PP 2408”	Pedraza 2408 (KJ788248, KJ788279, KJ788217)
*Satyria* sp.	Powell 9 (AY331882, AY331909, AY331953)
*Satyria* sp. nov. 1	Pedraza 2436 (KJ788251, KJ788283, KJ788220)
*Satyria* sp. nov. 2	Pedraza 1755 (KJ788252, KJ788282, KJ788221)
*Satyria latifolia* A.C.Sm.	Pedraza 1771 (KJ788249, KJ788280, KJ788218)
*Satyria leucostoma* Sleumer	Luteyn 15051 (AF382695, AF382780, AY331949)
*Satyria meiantha* Donn.Sm.	Luteyn 15236 (AF382696, AF382781, AY331950)
*Satyria panurensis* (Benth. ex Meisn.) Hook. f. ex Nied.	Luteyn 15247 (AF382697, AF382782, AY331951)
*Satyria pilosa* A.C.Sm.	Pedraza 2349 (KJ788250, KJ788281, KJ788219)
*Satyria polyantha* A.C.Sm.	Powell 83 (AY331881, AY331908, AY331952)
*Satyria vargasii* A.C.Sm.	Powell 75 (AY331883, AY331910, AY331954)
*Satyria ventricosa* Luteyn	Luteyn 15293 (AY331884, AY331911, AY331955)
*Satyria warszewiczii* Klotzsch	RBGE 781009 (AF382698, U61314, AY331956)
*Themistoclesia* sp. 1	Luteyn 15653 (m, KC175467, KC175456)
Thibaudia costaricensis Luteyn & Wilbur	Powell 16 (AY331887, AY331914, AY331963)
*Thibaudia densiflora* (Herzog) A.C.Sm.	Luteyn 15459 (AF382708, AF382790, AY331964)
*Thibaudia floribunda* Kunth	Luteyn 15090 (AF382709, AF382791, AY331966)
*Thibaudia inflata* Luteyn	Luteyn 15029 (AY331889, AY331916, AY331967)
*Thibaudia jahnii* S.F.Blake	Luteyn 15258 (AF382710, AF382792, m)
*Thibaudia litensis* Luteyn	Luteyn 15020 (AF382711, AF382793, AY331968)
*Thibaudia macrocalyx* J. Rémy	Luteyn 15444 (AY331890, AY331917, AY331969)
*Thibaudia martiniana* A.C.Sm.	Luteyn 15028 (AY331891, AY331918, AY331970)
*Thibaudia pachyantha* A.C.Sm.	Luteyn 15189 (AF382712, AF382794, AY331971)
*Thibaudia parvifolia* (Bentham) Hoerold	Luteyn 15212 (AF382713, AF382795, AY331972)
*Thibaudia tomentosa* Hoerold	Luteyn 15502 (AY331892, AY331919, AY331973)
*Vaccinium crenatum* (G.Don) Sleumer	Luteyn 14171 (AF382719, VCU89761, AF419742)

### Analytical methods

A multiple sequence alignment was produced using MUSCLE (Edgar 2004) and the model of sequence evolution was estimated with jModelTest 2 (Guindon and Gascuel 2003, Darriba et al. 2012). Maximum Likelihood (ML) estimation of the phylogenetic relationships was conducted using RAxML (Stamatakis 2006), employing one thousand replicates with stepwise random taxon addition, starting MP trees, the GTRGAMMA model of evolution, and 1000 bootstraps (BS) inferences. The best ML tree was visualized with FigTree 1.3.1 (http://tree.bio.ed.ac.uk/). The ML analyses were performed for each individual loci, partition (nuclear vs. plastid), and lastly, for the entire dataset.

## Results

**Figure 1.**
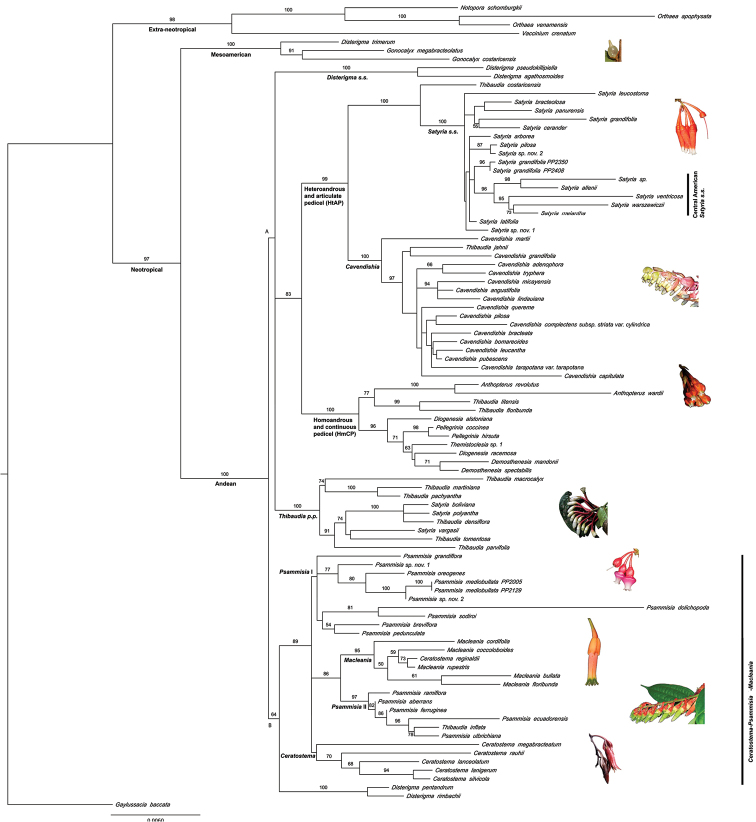
Phylogram of the best found Maximum likelihood hypothesis based on nuclear and plastid sequence data (ln*L*= -13984.363979). Bootstrap support values greater than 50% are shown in front of nodes.

The resulting topologies of the individual, partitioned (nuclear vs. plastid), and combined best ML trees are in general agreement (trees not shown) and no significant conflicts were detected. Thus, the results and discussion will focus on the best ML tree obtained from the combined analysis (Fig. 1). Only the more robust groups recovered by this analysis and those relevant to the placement of the new species from LONP will be discussed in detail.

The general topology of the most likely reconstruction (ln*L*= -13984.363979) is congruent with previous phylogenetic analyses of neotropical Vaccinieae. Here, some species from the Guyana Shield and the South American páramos are of extra-neotropical origin (98% bootstrap) and sister to a large neotropical clade. The neotropical clade (97% bootstrap) comprises a small Mesoamerican/Caribbean clade (100% bootstrap) sister to a large Andean clade (100% bootstrap), where the vast majority of the species are found. The Andean clade is divided into two major groups, named here A and B (Fig. 1).

Andean clade A (Fig. 1), is the larger of the two major groups, but it is the least supported (< 50% bootstrap). *Disterigma* *s.s.* (100% bootstrap) forms a tritomy with *Thibaudia* *p.p.* (including *Satyria* from the Central Andes, 100% bootstrap) and a clade that includes the Homoandrous and Continuous Pedicel (HmCP; equal stamens and pedicel continuous with the calyx; 100% bootstrap) and the Heteroandrous and Articulate Pedicel (HtAP; unequal stamens and pedicel articulated with the calyx; 99% bootstrap) clades. *Thibaudia* is polyphyletic with at least four independent origins within clade A and one species within clade B.

The HtAP group is the largest within clade A and *Cavendishia*, the most diverse of the neotropical genera (> 100 species), dominates it. *Cavendishia* is sister to the smaller *Satyria* *s.s.* (ca. 20 species). Sister to HtAP is the HmCP clade (*Anthopterus* + *Themistoclesia* + *Demosthenesia* + *Diogenesia* + *Pellegrinia* + *Thibaudia* *p.p.*), composed by putative genera that are very small or medium sized (5–30 species) (Fig. 1).

Andean clade B (64% bootstrap; Fig. 1) is made up of a clade previously denominated the Central Andes Segregated *Disterigma* (Pedraza-Peñalosa 2009, 2010a, 2010b) (100% bootstrap), sister to a tritomy (89% support) that contains the non-monophyletic *Ceratostema* Juss., *Macleania*, and *Psammisia*.

Overall, 73% of the genera of Andean origin (8 out of 11) for which more than one species was sampled were not monophyletic. The core *Disterigma* and *Satyria* clades have already been identified by previous studies, along with the species that need to be segregated from them (Powell and Kron 2003, Pedraza-Peñalosa 2009, 2010a); *Disterigma* *s.s.* was subsequently monographed (2010b). Therefore, *Disterigma* *s.l.* will not be further discussed. *Anthopterus* Hook. (100% bootstrap), *Demosthenesia* A.C.Sm. [including the type species, *Demosthenesia mandonii* (Britton) A.C.Sm., 71% bootstrap], and *Pellegrinia* Sleumer (98% bootstrap) are among the few Andean genera resolved as monophyletic. *Gonocalyx* Planch. & Linden (91% bootstrap) is another monophyletic group, but is of Mesoamerican origin (Fig. 1).

### *Satyria* *s.s.* clade

This HtAP subclade includes the majority of the sampled *Satyria* and is sister to *Thibaudia costaricensis* Hoerold. *Satyria* *s. s.* is strongly supported (100% bootstrap; Fig. 1); it includes the type of the genus, *Satyria warszewiczii*. *Satyria leucostoma* Sleumer forms a tritomy with the rest of *Satyria* *s. s.* The only well-supported groupings within *Satyria* *s.s.* are species pairs and a small subclade made up by taxa from Central America (96% bootstrap). Two of the sampled new species from LONP, *Satyria* sp. nov. 1 and *Satyria* sp. nov. 2, are placed within *Satyria* *s. s.*

### *Cavendishia* clade

In this clade (100% bootstrap; Fig. 1), *Cavendishia martii* (Meisn.) A.C.Sm. is strongly supported as sister to the rest. The only other well supported clade is the *Cavendishia micayensis*–*Cavendishia lindauiana* clade (≥ 94% bootstrap). *Thibaudia jahnii* S.F. Blake, from Venezuela, is included, and is sister to the Ecuadorean *Cavendishia grandifolia* Hoerold (support weak).

### *Thibaudia* *p.p.* clade

Within this clade (100% bootstrap; Fig. 1), *Thibaudia macrocalyx*–*Thibaudia pachyantha* form a cluster (74% bootstrap) sister to a clade (91% bootstrap) that contains three species of *Satyria* mixed with other species of *Thibaudia*.

### *Psammisia*–*Ceratostema*–*Macleania* clade

Within the Andean clade B, *Psammisia* is split in two clusters (Fig. 1). The largest of the groupings, *Psammisia* I clade (< 50% bootstrap), is part of a tritomy that also contains *Ceratostema* (< 50% bootstrap) and the *Psammisia* II + *Macleania* clade (86% bootstrap). *Psammisia* II (97% bootstrap) (including *Thibaudia inflata* Luteyn) is sister to *Macleania* (96% bootstrap). Neither *Ceratostema* nor *Macleania* are monophyletic as *Ceratostema reginaldii* (Sleumer) A.C.Sm. is derived within *Macleania*. The other two sampled new species from LONP are resolved within *Psammisia* I.

## Discussion

The two main groupings within the Andean clade, clades A and B (Fig. 1), also appear in previous studies with large generic coverage (see Kron et al. 2002, Pedraza-Peñalosa et al. 2013). However, these studies differ in their generic sampling and the groups in which they disagree are either poorly supported or sampled, making detailed comparisons difficult. The following are the most significant findings.

### HtAP clade

This strongly supported group (99% bootstrap; Fig. 1) is among the largest Andean Vaccinieae subclades with an estimated 170 species, most of them *Cavendishia*. Heteroandrous taxa are very distinctive within Vaccinieae and are characterized by having strongly unequal stamens in which the filaments and/or anthers of adjacent stamens (each corresponding to one of the two staminal whorls) alternate in length. When there are differences in anther size, dimorphism can be also expressed in anther shape (*Satyria* *s.s.*) and orientation of the tubule aperture (*Satyria* *s.s.*, *Cavendishia*). However, differences in this latter feature are poorly documented in species descriptions and are at times slight, and neither its extent nor its consistency is clear.

A close evolutionary relationship between *Satyria* *s.s.* and *Cavendishia* was first proposed by Smith (1932) who suggested that even though the genera are distinct, they form a separate group within Vaccinieae having staminal dimorphism in common. However, the nature of the dimorphism is quite different in each group, as pointed out below, and it should be further examined. Smith included the also dimorphic *Orthaea* within this group, but Andean species of *Orthaea* were not included in this analysis as it is currently being studied in detail (revision in preparation by N. R. Salinas).

Lastly, the HtAP species are characterized by having the pedicel articulate with the calyx (seen as a constriction at the point of attachment), a homoplasic feature that is traditionally used as part of the key characters useful to tell genera apart.

### *Satyria* *s.s.* clade

*Thibaudia costaricensis* is sister to *Satyria* *s.s.*, a clade that is in agreement with the molecular circumscription of *Satyria* by Powell and Kron (2003). Both taxa have connate filaments (Fig. 1). *Satyria* *s.s.* species have in common markedly alternately unequal stamens with rigid dimorphic anthers, the longer of which have flaring tubules that are often ornamented or recurved into hooks; their filaments are equal in length (Powell and Kron 2003). Powell and Kron (2003) sampled 11 out of the 22 then recognized species and noted that the Colombian *Satyria* (10 species, 9 of them endemic) needed to be included in future studies to better elucidate evolutionary patterns. Seven Colombian species, including two new to science, are added to this analysis for a total of 18 *Satyria* *s. l.*

Fifteen morphospecies are placed within *Satyria* *s.s.*, mostly South American (Fig. 1). However, despite the increased taxonomic coverage, support at the basal nodes is weak and the only well-supported major subclade is that of the Central American species (*Satyria allenii*–*Satyria meiantha*, 96% bootstrap). The derived position of these Central American species indicate they are the result of relatively recent dispersals from a South American ancestor. However, in the case of *Satyria panurensis* (Benth. ex Meisn.) Hook. f. ex Nied., a species found from Mexico to southern Bolivia (including the Guianese Shield to the east) and not included within the Central American clade of *Satyria* *s.s.*, the direction of the migration of is not clear.

Although the new species are endemic to the same general region, have similar corolla colors and shape, and are the only *Satyria* *s.s.* known to have an ornamented calyx (winged and/or lobed), they are not sister species. *Satyria* sp. nov. 1, sister to a clade containing species from both Central and South America, is easily differentiated from all other *Satyria* *s.s.* because of its pseudoverticillate leaves. *Satyria pilosa* A.C.Sm., another newly sequenced species, present in Antioquia but also beyond, being endemic to the greater Chocó biogeographic region, is sister to *Satyria* sp. nov. 2 (87% bootstrap).

Some of the molecular-based relationships here obtained using a larger sampling of *Satyria* *s.s.* do not agree with some of the taxonomic rearrangements of a recent monographic study (Powell 2005). Although the proposed changes have not been formally published, the unpublished names and combinations have already appeared in taxonomic and record-based public databases, as well on annotations of herbarium specimens from several American and European herbaria. Hence the comments below.

*Satyria warszewiczii* is a species thought to be confined to Central America (southern Mexico to Panama), with a broad altitudinal gradient [(100–)300–2500 m] and consequently morphological variation (Luteyn and Wilbur 2005). Based on Principal Components Analyses of the morphological variation, Powell (2005) suggests that 11 species, mostly from South America, are indistinguishable from it and therefore should be synonymized.

Molecular data for *Satyria latifolia* A.C.Sm. and *Satyria ventricosa*, as well as two morphospecies of the variable *Satyria grandifolia* (Colombia–Peru) from NW Colombia, all putative synonyms of *Satyria warszewiczii* according to Powell (2005), were sampled. A third collection of *Satyria grandifolia* from SW Colombia, which was used in previous phylogenetic analyses (see Powell 2005, Powell and Kron 2003), was also analyzed.

The Central American *Satyria meiantha*, *Satyria warszewiczii*, and *Satyria ventricosa* form a clade (95% bootstrap; Fig. 1), in agreement with Powell’s taxonomic proposal, and they may as well be conspecific, although terminal branch lengths are very long. However, they are more closely related to *Satyria allenii* A.C.Sm., which was considered by Powell (2005) a distinct species, than to either *Satyria latifolia* or *Satyria grandifolia*.

On the other hand, the two newly sequenced specimens of *Satyria grandifolia* from NW Colombia form a well-supported clade sister to the Central American *Satyria* *s. s.*, but with little support, while the *Satyria grandifolia* from SW Colombia is placed with species of extra-Andean distribution, although again with little support (Fig. 1). As for *Satyria latifolia*, this species is part of a poorly supported tetratomy and is not immediately related to either *Satyria warszewiczii* or any accession of *Satyria grandifolia*, as hypothesized. Moreover, there are no big differences between the samples of *Satyria grandifolia* from NW Colombia, whereas considerable changes have accumulated on the branches and terminals in their sister group, *Satyria warszewiczii* and relatives.

Altogether, the results suggest that the *Satyria grandifolia* from NW and SW Colombia are not conspecific. The herbarium vouchers of the *Satyria grandifolia* from NW Colombia were collected in the same biogeographic region (Chocó) where the type species was procured, about 200 km from the type locality. To rule out contamination of our sample, some molecular markers were independently re-sequenced and identical results were obtained. The herbarium voucher of the *Satyria grandifolia* from SW Colombia (*Luteyn 15204*) was collected much farther away from the type locality, but still within the Chocó biogeographic region. Unfortunately, because *Luteyn 15204* does not have flowers (only fruits), it is not possible to reassess its taxonomic identity.

In the same general area of *Luteyn 15204* there are specimens very similar to those from NW Colombia, however, others have floral and vegetative characteristics that subtly diverge from them and which have not been observed in other studied *Satyria grandifolia* collections. Without doubt Powell was right at pointing out that species delimitation within *Satyria* *s.s.* is complicated and that more fieldwork in western Colombia was advised.

It was also (Powell 2005) suggested that *Satyria arborea* A.C.Sm., endemic to Colombia, should be synonymized with *Satyria allenii*, endemic to Panama. However, in this analysis they are not sister species, with *Satyria allenii* placed at a more derived position within a well-supported clade that includes other Central American *Satyria* *s. s.* (Fig. 1). The newly sequenced *Satyria arborea* was collected relatively close to the type locality and it is easily differentiated from *Satyria allenii* by anther length (about twice as long in *Satyria arborea*). Anther length may be one of the best characters, beyond geographic distribution, to differentiate among these species, as other traditionally used features such as length of the petiole and pedicel and life form have been shown to be inadequate (Powell 2005).

### *Cavendishia* clade

Filaments and anthers of adjacent stamens are of different lengths in *Cavendishia*, a diagnostic character that has been invoked by most of the taxonomic classifications of the neotropical Vaccinieae of the last century (Luteyn 1983, Smith 1932, Sleumer 1941). However, the *Cavendishia* clade also includes *Thibaudia jahnii* (but < 50% bootstrap) toward its base (Fig. 1), a taxon that lacks the characteristic heteroandrous morphology of the genus *Cavendishia* and of the entire HtAP clade. Besides sharing free stamens, there are no other apparent morphological characters that satisfactorily explain the placement of *Thibaudia jahnii* within this clade

*Cavendishia* has approximately 130 species, most of which are native/endemic to Colombia. The 15 species of *Cavendishia* here analyzed include representatives from the two currently recognized subgenera of *Cavendishia*: *Chalmydantha* and *Cavendishia*, as well as of four of the five sections of subgenus *Cavendishia* (*Foreroa* is missing), all but one of the series of section *Cavendishia* (*Uniflorae* is missing), and all but series Lactiviscidae of section Engleriana (see Table 1). Unfortunately, none of the subgenera, sections, or series of *Cavendishia* is resolved as monophyletic. However, it must be cautioned that the phylogenetic relationships recovered within *Cavendishia* are too poorly supported to draw strong conclusions and a more extended taxonomic/molecular sampling of it is needed.

From a more general point of view, taxa present in Central America have a more derived position, but unlike *Satyria* *s.s.* in which Central American species are clustered together, in *Cavendishia* the Central American species are dispersed throughout the clade [*Cavendishia lindauiana* Hoerold, *Cavendishia quereme* (Kunth) Benth. & Hook. f., *Cavendishia pubescens* (Kunth) Hemsl., *Cavendishia capitulata* Donn.Sm., *Cavendishia bracteata* Ruiz & Pav. ex J.St.-Hil.].

### HmCP clade

The relationships recovered for this clade (Fig. 1) are consistent with those found by Pedraza-Peñalosa et al. (2013). Taxa in this clade all have equal stamens and their pedicels are continuous with the calyx, unlike the HtAP clade, the sister group. That being said, the sampled species of *Demosthenesia* A.C.Sm. have slightly unequal filaments, but not as markedly as in the HtAP clade.

The HmCP clade unites groups with diverse morphologies. The only monophyletic genera within it are relatively small (up to 12 spp.) and have contrasting geographic patterns: *Anthopterus* is widely distributed in the neotropics, while *Pellegrinia* and *Demosthenesia* are both endemic to a small area of the Peruvian and Bolivian Andes (Fig. 1). However, many subclades within HmCP are well supported and the two largest stand out. The four species of the *Anthopterus revolutus*–*Thibaudia floribunda* clade (77% bootstrap) have in common winged or angulate calyces and corollas, whereas the seven species of the *Diogenesia alstoniana*–*Demosthenesia spectabilis* clade (96% bootstrap) in contrast all have terete calyces and corollas.

Only one species of *Themistoclesia* Klotzsch was included in this analysis. However, *Themistoclesia* with articulate calyces have recently been described, but unfortunately, none of them was available for sequencing. These taxa also have other characteristics not previously thought to occur in the genus and it has been hypothesized they may represent a geographically and morphologically distinct clade (Pedraza-Peñalosa and Luteyn 2010). Thus, future analyses should sample in more detail the morphological diversity of *Themistoclesia*, as well as that of other members of the HmCP clade.

### *Thibaudia* *p.p.* clade

All species in the *Thibaudia* *p.p.* clade have pedicels articulated with the calyx; it is precisely the presence of such articulation that initially defined Thibaudia section Eurygania, currently a synonym of *Thibaudia*. However, the staminal characters that seem to be important in defining other larger clades are absent here. This analysis supports Powell and Kron’s (2003) assessment that species of *Satyria* in this clade, all endemic to S Peru and N Bolivia, should be segregated from *Satyria* *s.s.* (Fig. 1). The anther tubules of these *Satyria* do not diverge much distally, their sides being more parallel, and their tips lack ornamentations, so differing from *Satyria* *s.s.* (Powell 2005). The *Thibaudia* and *Satyria* species that cluster together all have equal or slightly unequal stamens and connate filaments.

On the other hand, although the *Thibaudia macrocalyx*–*Thibaudia pachyantha* subclade (74% bootstrap) also includes species with equal stamens, their filaments are free. Other characters that are alos shared by some of the members of the *Thibaudia* *p.p.* are thick corolla and calyx limb, anthers with poor distinction between tubules and thecae, thecae papillose and dehiscence by ventral clefts.

### *Psammisia*–*Ceratostema*–*Macleania* clade

*Psammisia* *s.l.* contains species with terete to winged calyces; fused, free or coherent staminal filaments; short to long corollas; and pinnate or plinerved laminas. It also includes perhaps the greatest variety of corolla shapes of any neotropical Vaccinieae (tubular, obconic, urceolate, turbinate and depressed, hemispheric); and also a great variety of corolla colors (yellow, magenta, vermilion, dark wine, red, white, green, etc.) and color combinations (solid, bicolor, multicolor).

The unifying staminal features of *Psammisia* *s.l.* include stout anthers, free tubules, and connectives, the region where filaments adhere to the anthers, that are 2-spurred, alternately spurred (i.e. only one staminal cycle is spurred), or rarely unspurred. It is precisely because of the presence of spurs that Smith (1932) linked *Psammisia* to a group of *Macleania* whose connectives are faintly thickened distally. These thickened connectives were interpreted as a step toward the spurred condition observed in some *Psammisia* *s. l.* A morphological connection between members of *Psammisia* and *Macleania* is suggested by these results, as *Psammisia* *s.l.* is resolved in two clades, one of them sister to *Macleania*. However, the morphological basis of this relationship it is not yet known. As for the second grouping of *Psammisia* *s.l.*, it is part of a more basal tritomy. Unfortunately, because taxon sampling is still inadequate and the type species of the genus was not available for sequencing, it is uncertain which *Psammisia* clade will retain the name.

### *Psammisia* I clade

Most of the sampled *Psammisia* *s.l.* are found within the *Psammisia* I clade (*Psammisia grandiflora*–*Psammisia pedunculata*, < 50% bootstrap), which is dominated by species from the N Andes (Fig. 1). *Psammisia dolichopoda* A.C.Sm. is the only species that is also present in Central America. However, ongoing studies suggest that it has considerable morphological variation and may represent more than one species (Pedraza-Peñalosa pers. obs.).

Two of the new species from LONP are placed here, within a clade (77% bootstrap) dominated by Colombian taxa; the only exception is the rare *Psammisia oreogenes* Sleumer, which was earlier thought to be exclusive to Ecuador but is now known to also occur in the Colombian portion of the Chocó biogeographic region (Fig. 1). The new species from LONP portray morphological characters that are either unusual in *Psammisia* *s.l.* (e.g., *Psammisia* sp. nov. 1 has large leaves with pinnate venation) or previously unknown (i.e., *Psammisia* sp. nov. 2 is the only known taxon with pseudoverticillate leaves). In general, the novel taxa are morphologically different and molecular sequence data indicate they are not immediately related.

It is difficult to find unifying morphological characters for *Psammisia* I. Moreover, the clade lacks support and has a tritomy at its base. Within it, only the *Psammisia* sp. nov. 1–*Psammisia* sp. nov. 2 subclade has moderate support (77% bootstrap). All its species share chartaceous to subcoriaceous leaves, pinnate venation, racemes with short rachises typically less than 1.6 cm long making the inflorescences look fasciculate, and medium sized corollas 8–22 mm long.

### *Psammisia* II

Sequence data shows that *Psammisia* II taxa are more closely related to *Macleania* than to other congeneric species (Fig. 1). Unifying characteristics of the *Psammisia* II group comprise coriaceous leaves, plinerved venation, racemes with usually conspicuous rachises (1–26 cm long), and long corollas (17–40 mm). The inclusion of *Thibaudia inflata* within *Psammisia* II in a relatively derived position, deserves further scrutiny as its pedicel and calyx are continuous, whereas in most *Macleania*, and in all *Psammisia* *s.l.* it is articulated.

Furthermore, other morphological features seem to also help to differentiate between the *Psammisia* I and II clades. The leaves of *Psammisia* II have laminar glands at the base of the abaxial side, while the leaves of *Psammisia* I do not have basal glands, or when laminar glands are present, they are then spread through the entire abaxial surface (e.g., *Psammisia sodiroi*, see Pedraza-Peñalosa et al. 2013). Anther morphology also seems to be helpful, with *Psammisia* II possessing well-developed tubules of a diameter similar to that of the theca, while in *Psammisia* I tubules are smaller and narrower, of a basal diameter only a fraction of the theca width.

### *Macleania* clade

*Macleania* is sister to *Psammisia* II and there is good support for this clade and this relationship; the former clade includes *Macleania floridunda* Hook., the type species of the genus (Fig. 1). Because of its morphological range, Smith (1932) considered *Macleania* a coherent genus from which other genera were in the process of being derived. However, the subgenera *Aponema* and *Macleania*, which were thought to be morphologically well delimited, are not monophyletic. Moreover, although most sampled *Ceratostema* form a clade (but unsupported) placed within the basal tritomy of the *Psammisia*–*Ceratostema*–*Macleania* clade, *Ceratostema reginaldii* is resolved within *Macleania*.

As for *Ceratostema*, although as currently circumscribed is morphologically recognizable, it is not monophyletic and the sampled species fail to form a well supported clade. *Ceratostema* was one of the first genera to be erected, and because Ericaceae is a predominantly montane group with a high number of endemics, it is not difficult to imagine the challenges earlier taxonomists faced to procure sufficient specimens for their studies. Limited collections and field observations led to problematic generic classifications. Consequently, not only many species have been transferred out of *Ceratostema*, but also entire genera have been segregated from within it such as *Demosthenesia* and *Pellegrinia*. Both genera are here resolved as monophyletic and not closely related to any sampled *Ceratostema*. Unfortunately, both taxon (16 out of ca. 75 spp.) and molecular sampling are still insufficient to better discern the evolutionary relationships between *Macleania* and *Ceratostema*.

## Conclusions

With the study of the undersampled Colombian taxa, a critical component of neotropical Vaccinieae, a more complete picture of the complexity of the phylogenetic relationships within the Andean Vaccinieae has emerged.

The molecular results suggest that the observed diversity of neotropical Vaccinieae is mostly due to the diversification of several clades of Andean origin that do not necessarily correspond to the current taxonomic classification. Several genera that have been traditionally broadly defined are resolved as non-monophyletic and it seems likely they could be further split into more discrete groups. All this points to the need for broad-scale comparative anatomical and morphological studies to reevaluate homologies, synapomorphies and clade support in general.

Indeed, although a cladistic analysis of morphological characters is premature at this point, it was still possible to identify morphological characters that seem to differentiate among some major clades and subclades. Most notable are the HtAP and HmCP clades, both strongly supported molecularly and easily diagnosable morphologically – they reflect the diversity of arrangements and morphologies of the stamens and flowers of Vaccinieae. However, although morphological characters may diagnose clades in one part of the tree, they may vary within a clade in another part of the tree. Such is the case of the fusion of staminal filaments, a character diagnostic for *Satyria* *s.s.*, the HmCP, and *Cavendishia* clades, but which is variable within the well-supported *Macleania* clade.

This analysis unequivocally places the newly discovered Colombian Vaccinieae within *Satyria* *s.s.* and the *Psammisia* I clade, 2 species in each clade, but not closely related to each other within their respective clades.

The small *Satyria* *s.s.* is a complex genus with species limits that are hard to elucidate. It is now clear that many morphological characters previously thought to differentiate species, especially those based on the scant voucher specimens available to earlier workers, do not work in the light of today’s better documented intraspecific variation. Molecular results suggest that continuous characters (i.e., size) include informative data and can be used to distinguish species. Fieldwork in LONP suggests that characters that are not reported in herbarium labels and that cannot be recognized in herbarium specimens may be useful to work out species hard to differentiate (i.e. tridimensional shape of calyx, corolla, fruits, seed and embryo color) and these characters need to be reported on a regular basis.

Taxon sampling was nearly doubled in this analysis when compared to previous studies dealing with Andean species and robust monophyletic groups such as, *Anthopterus*, *Demosthenesia*, *Gonocalyx*, *Pellegrinia*, and *Satyria* *s. s.*, were identified within the Andean clade, although admittedly the first four genera included only two species each. However, *Gonocalyx*, present in both Central and South America, may be of Mesoamerican origin. Clearly, more exhaustive analyses are necessary to fully resolve intergeneric relationships, and even the monophyletic groups here discerned (with exception of *Satyria* *s. s.*) need better representation. Further phylogenetic work is obviously needed for the large and non-monophyletic *Psammisia* *s.l.*, *Macleania*, *Thibaudia* *s.l.* and *Ceratostema*, accompanied of field and herbarium work.

Lastly, it is also important to increase the representation of Central American species in order to further explore diversification and colonization of that region. In these results, there is evidence of multiple dispersals of Andean Vaccinieae to Central America and of at least one radiation within Central America (Central American *Satyria* *s.s.*) of a genus of Andean origin.
